# MicroRNA‐223 alleviates inflammatory response in renal ischemia‐reperfusion injury by targeting NLRP3

**DOI:** 10.1002/kjm2.12883

**Published:** 2024-09-06

**Authors:** Jun Ye, Xiaoli Tang, Ming Li, Yutian Liao, Yiqian Zeng, Furong Tang, Eryue Qiu

**Affiliations:** ^1^ Trauma Center, Zhuzhou Hospital Affiliated to Xiangya Medical College Central South University Zhuzhou Hunan China; ^2^ Department of Emergency, Zhuzhou Hospital Affiliated to Xiangya Medical College Central South University Zhuzhou Hunan China

**Keywords:** MiR‐223, NLRP3, renal inflammation, renal ischemia‐reperfusion injury, TLR4

## Abstract

We investigated the potential correlation between miR‐223 and NAcHT, LRR, and PYd domain‐containing protein 3 (NLRP3) in the context of renal ischemia‐reperfusion injury (RIRI), which is a leading cause of acute renal failure with significant mortality rates. Additionally, miR‐223 has been implicated in renal inflammation, further highlighting its relevance to this study. C57BL/6 male mice were used as RIRI models. After successful modeling, pathological examinations and serum creatinine and miR‐223 levels were tested. Pro‐inflammatory cytokine (IL‐1β, IL‐6, IL‐8, NLPR3, TLR4) expression was detected in mice by western blot (kidney tissue) and enzyme‐linked immunosorbent assay (serum). HK‐2 cells were used for in vitro experiments. A hypoxia/reoxygenation (H/R) model was used, and miR‐223 and pro‐inflammatory cytokine levels were detected using PCR and western blot assays, respectively. A dual‐luciferase reporter assay was conducted to confirm the binding of miR‐223 to NLPR3. Next, NLRP3 was knocked down to determine whether the anti‐inflammatory function of miR‐223 is dependent on NLRP3. MiR‐223 expression was lower in RIRI mice than in the sham operation group. The level of miR‐223 negatively correlated with serum creatinine levels and the severity of tubule injury. Increased proinflammatory cytokine levels in RIRI mice were observed. In vitro, miR‐223 alleviated the inflammatory response in H/R treated cells by inhibiting proinflammatory cytokines. Dual‐luciferase reporter and western blot assays confirmed the binding of miR‐223 to NLRP3. NLRP3 knockdown reversed the anti‐inflammatory effects of miR‐223 in HK‐2 cells. MiR‐223 plays an anti‐inflammatory role in RIRI by targeting NLRP3 to repress pro‐inflammatory factors.

## 1 INTRODUCTION

Renal ischemia‐reperfusion injury (RIRI) is one of the main causes of acute kidney failure and is associated with organ failure and high mortality.^1^, ^2^ RIRI occurs in severe trauma, thrombosis, and kidney transplantation. RIRI refers to a process in which blood supply to the kidney is suddenly reduced, and after a period of ischemia, the blood supply to the kidney recovers; inflammatory responses and cellular apoptosis occur.^3^ Pro‐inflammatory substances accumulate in the kidneys and cause organelle damage. If injury progresses, cell death is initiated, which can trigger an immune response in the kidney. Because of its large blood supply and urine generation, the kidney is very sensitive to RIRI, and delayed treatment or neglect of RIRI usually leads to a poor prognosis. Hence, the early diagnosis and efficient treatment of RIRI are important.

MicroRNAs (miRNAs) are small non‐coding RNAs that post‐transcriptionally modulate gene expression.^4^ MiRNAs can bind to a target gene to regulate its expression. Interactions between miRNAs and their target genes form a complicated network involved in cellular metabolism, apoptosis, and immune responses. Recent investigations have focused on miR‐223 in lung IRI and lipopolysaccharide (LPS)‐induced acute kidney injuries owing to its protective functions.^5^, ^6^ MiR‐223 can protect cardiomyocytes from fibrosis and radiation‐induced toxicity^7^, ^8^; it is widely involved in multiple‐organ inflammation and oxidative stress responses. NAcHT, LRR, and PYd domain‐containing protein 3 (NLRP3) are vital for inflammatory responses in various diseases.^9^ TLR4 is a pivotal factor in the TLR4/NF‐κB signaling pathway. Activated NF‐κB can translocate from the cytoplasm to the nucleus to further activate NLRP3. The assembly of the NLRP3 inflammasome can release several inflammatory cytokines like IL‐1β. A previous study demonstrated that miR‐223 can bind to NLRP3 to suppress inflammation in lung disease.^10^, ^11^ However, the role of miR‐223 in RIRI remains unclear. In this study, we attempted to elucidate the anti‐inflammatory role of miR‐223 in RIRI using both in vivo and in vitro studies.

## 2 MATERIALS AND METHODS

### 2.1 Animal models

C57BL/6 male mice weighing 150–170 g were purchased from Hangzhou Medical College. This study was approved by the Ethics Committee (No. 2022743). All animal experiments were performed in compliance with *Guide for the Care and Use of Laboratory Animals*. All the mice were acclimatized to the laboratory environment prior to randomization. The mice were randomly allocated to sham operation and RIRI groups. All the animals were anesthetized with sevoflurane using an animal anesthesia machine (VMP, MATRX) in the supine position. The RIRI models were established briefly as follows: after anesthesia, a ventral median incision of 3–4 cm was made, and a warm saline‐soaked dressing was used to cover the organs after opening the abdomen. Subsequently, a nephrectomy of the right kidney was performed. The left renal pedicle was clamped using a vascular clamp for 45 min and then released. The initial screening criteria for successful models were as follows: 5 min after release from the clamp, the color of the kidney changed from violet‐black to brick‐red; otherwise, the model failed. The abdomen was then closed with sutures. The sham operation group received anesthesia and underwent opening and closing of the abdomen. After 24 h of observation, blood samples were collected from the retro orbital vein (50 μL) of anesthetized (sevoflurane) mice weighing 150 ± 20 g for creatinine testing. The levels of creatinine (Cr) and blood urea nitrogen (BUN) were measured using creatinine (Abcam, ab65340) and urea assay kits (Abcam, ab83362), respectively, following the manufacturer's instructions. The mice were sacrificed, and the left kidney was harvested for hematoxylin and eosin staining and other studies. Advanced sacrifice was performed if mice reached humane endpoints, including moribund symptoms, difficulties in water or food intake, or severe loss of fur. Tubule injury severity was evaluated as follows: 0, no damage; 1, <25%; 2, 25%–50%; 3, 50%–75%; and 4, >75%.^12^

### 2.2 Cell culturing and transfection

Human proximal tubular cells (HK‐2 cells) were purchased from Pricella (Wuhan, China). HK‐2 cells were cultured in MEM (PM150410; Pricella, Wuhan, China) and maintained at 37°C in a humidified incubator containing 5% carbon dioxide. For hypoxia and re‐oxygenation (H/R) experiments, HK‐2 cells were cultured in 89% DMEM (Hakata, Chuan Qiu Biotechnology, Shanghai, China) + 10% FBS (Hakata, Chuan Qiu Biotechnology, Shanghai, China) + 1% penicillin–streptomycin solution (Hakata, Chuan Qiu Biotechnology, Shanghai, China) and incubated in 94% N_2_, 5% CO_2_, and 1% O_2_ (37°C) for 12 h. After 12 h of incubation, HK‐2 cells were placed in a normal incubator.

miR‐223 mimic (5′‐UGUCAGUUUGUCAAAUACCCCA‐3′) and NC (Ctr; 5′‐UGUCAGUUUGUCAAAUACCCCA‐3′) were transfected using Lipofectamine® 3000 (Invitrogen; Thermo Fisher Scientific, USA). All small nucleic acids were purchased from GenePharma (Shanghai, China) at a final concentration of 20 nM. Cells were harvested for further assays 48 h after transfection.

To decrease NLRP3 expression, HK‐2 cells were cultured in 6‐well plates and harvested after reaching 80% confluency. The cells were incubated for 24 h with 100 nmol/L NLRP3‐siRNA and control siRNA using Lipofectamine® 3000 (Invitrogen; Thermo Fisher Scientific, USA) according to manufacturer's instructions.

The sequences were siRNA 1 5′‐GAAAUGGAUUGAAGUGAAAdTdT‐3′ (sense) and 5′‐UUUCACUUCAAUCCAUUUCdTdT‐3′ (antisense) and siRNA 2 5′‐GGAUCAAACUACUCUGUGAdTdT‐3′ (sense) and 5′‐UCACAGAGUAGUUUGAUCCdTdT‐3′ (antisense) for NLRP3; the negative control siRNA (NC group) was purchased from Shanghai GenePharma (Shanghai, China).

### 2.3 Quantitative real‐time polymerase chain reaction (RT‐qPCR) assay

RNA extraction of the cells was performed with an MiPure Cell/Tissue miR kit (Vazyme, Nanjing, China); the reverse transcription was carried out by BeyoRT™ II cDNA (Beyotime, China) following the manufacturer's instructions. The following primer sequences were used: miR‐223 forward: 5′‐CCGCTCGAGGAGCTTCCAGCTGAGCACTGGG‐3′, reverse: 5′‐CGACGCGTTATTGCGCCCCCATCAGCACT‐3′; NLRP3 forward: 5′‐CCATCGGCAAGACCAAGA‐3′, reverse: 5′‐ACAGGCTCAGAATGCTCATC‐3′; human U6 forward primer: 5′‐CTCGCTTCGGCAGCACA‐3′, reverse primer: 5′‐AACGCTTCACGAATTTGCGT‐3′. The normalization reference was U6.

The ΔΔCt method was used for calculation of the gene expression. The PCR detection system used was the ABI QuantStudio 5 (Thermo Fisher Scientific, A28569).

### 2.4 Western blot assay

Proteins were extracted from tissues and cells using RIPA Lysis Buffer (Epizyme, China). Next, the extracted proteins were separated by 10% polyacrylamide gel electrophoresis. Proteins were transferred to a polyvinylidene fluoride (PVDF) membrane (Millipore, Billerica, MA, USA). The cells were then blocked with Tris‐buffered saline. Tween (TBST) buffer contained 0.05% Tween‐20 and 5% nonfat dry milk (Biosharp, Hefei, China), and the protein‐containing membrane was incubated with corresponding primary antibodies at 4°C overnight. The following antibodies were used in the study: anti‐IL‐1 beta antibody (RM1009, ab283818), anti‐IL‐6 antibody (EPR23819‐11, ab259341), anti‐IL‐8 antibody (EPR26511‐74, ab289967), anti‐NLRP3 (EPR23073‐96—BSA and Azide free ab272702), anti‐TLR4 antibody (ab218987), anti‐NF‐kB antibody (E379, ab32536), anti‐ASC antibody (EPR28201‐61, ab307560), anti‐Caspase‐1 antibody (ab138483), and anti‐GAPDH antibody (6C5—Loading Control ab8245). The membrane was incubated with horseradish peroxidase (HRP)‐conjugated AffiniPure goat anti‐rabbit IgG (H + L) for 2 h. The membranes were then washed and treated with freshly prepared ECL luminescent working solution (Beyotime, P0018S). The protein bands were detected and calculated using ImageJ software.

### 2.5 Dual luciferase reporter assay

We searched Targetscan (www.targetscan.org) to find a potential binding site of miR‐223 and NLRP3 3′‐UTR. 3′‐UTR sequences of NLRP3 containing wild‐type (wt) and mutant (mut) of miR‐223 binding sties were purchased from GenePharma (Shanghai, China). The vectors were co‐transfected with anta‐miR‐223 and control groups into HK‐2 cells and incubated for 48 h. A Dual‐Glo® Luciferase Assay System (Promega, Madison, USA) was used to detect the luciferase activity following the manufacturer's protocol. Luciferase activity was used for internal normalization.

### 2.6 Immunofluorescence assay

Slides containing the experimental cells were washed thrice with PBS and fixed with triformol. The slides were then blocked with serum at room temperature. Next, the slides were incubated with the primary antibody (anti‐NLRP3 [EPR23073‐96]—BSA and azide‐free [ab272702]) and secondary antibodies. Finally, after staining with DAPI, the slides were sealed with anti‐fluorescence quencher (Beyotime, P0123‐5 mL), observed, and analyzed by microscopy.

### 2.7 Enzyme‐linked immunosorbent assay

We measured the relative expression of inflammatory cytokines in the kidneys of the animals using enzyme‐linked immunosorbent assay (ELISA). Briefly, the experimental steps were as follows: pre‐cooled normal saline was used to wash the harvested kidney three times, and the connective tissues was cut and weighed. Kidneys were cut into pieces and humidified in PBS. The kidneys were sonicated using an ultrasonic homogenizer and centrifuged. The supernatant was used for ELISA. The corresponding ELISA kit was used according to the manufacturer's instructions. The detailed ELISA kits used were as follow: rat interleukin 1β (IL‐1β) ELISA Kit (CUSABIO, CSB‐E08055r, China), rat interleukin 6 (IL‐6) ELISA Kit (CUSABIO, CSB‐E04640r, China), rat IL‐8 ELISA kit (ZCi Bio, ZC‐36406), rat NLRP3 ELISA kit (ZCi Bio, ZC‐54271, China), and rat toll‐like receptor 4 (TLR4) ELISA kit (CUSABIO, CSB‐E15822r, China).

### 2.8 Statistical analysis

Data are shown as mean ± standard deviation (SD) of three independent experiment repeated three times. GraphPad Prism 7 (San Diego, CA, USA) was used to analyze the data. Based on the principle of statistical analysis, Student's *t*‐test and chi‐square test were used. Statistical significance was set at *p* < 0.05.

## 3 RESULTS

### 3.1 MiR 223 was decreased in RIRI mice and negatively correlated with serum Cr and BUN

**FIGURE 1:**
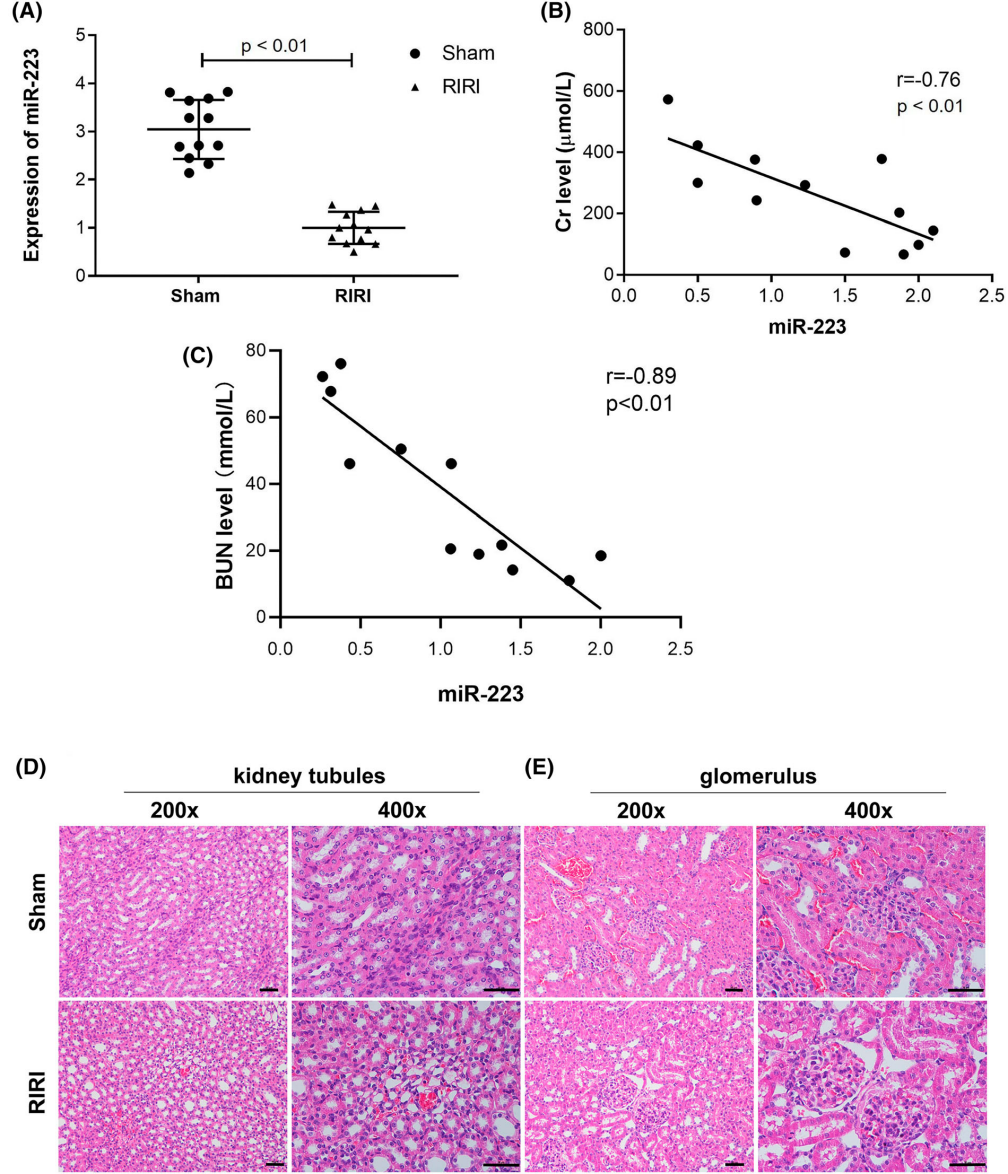
Level of miR‐223 in sham operation and RIRI mice using plasma samples at the 0 h mark via RT‐qPCR (A); correlation of creatinine and blood urea nitrogen levels with miR‐223 in RIRI mice using plasma samples at 0 h mark (B and C); HE staining of the RIRI kidney in mice. Micrograph of kidney tubules in sham‐operation group and RIRI group (D); micrograph of glomerulus in sham‐operation group and RIRI group (E). Data are shown as mean ± SD.

There were 6 mice in the sham operation group and 12 in the operation group (RIRI). Harvested kidneys were used for RNA extraction and PCR. The miR‐223 levels were lower in the RIRI group than in the sham operation group (Figure 1A). We analyzed the serum Cr and BUN levels of mice and found that they were negatively correlated with the levels of miR‐223 (Figure 1B,C). All mice in the RIRI group were successfully modeled, with a mean tubule injury severity score of 3 points (sham operation group). Figure 1D,E shows typical H&E staining of RIRI mice and the sham operation group. As mentioned above, we found that the miR‐223 level was significantly lower in the RIRI group than in the sham operation group. The level of miR‐223 was even lower in the more injured mice (according to pathological evaluation under a microscope), with higher serum creatinine and BUN levels.

### 3.2 The different expression levels of miR‐223 and NLRP3 in the kidneys of RIRI mice over 24 h

**FIGURE 2:**
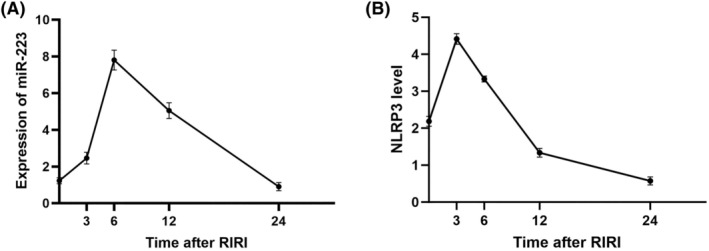
Expression levels of miR‐223 and NLRP3 in the kidneys of RIRI mice at various time points (0, 3, 6, 12, and 24 h) post‐RIRI induction via RT‐qPCR. The level of miR‐223 gradually increased, peaking at 6 h before gradually declining by the 24‐h mark (A). NLRP3 levels increased at 3 h and decreased significantly at 6 h, indicating an inverse relationship between NLRP3 and miR‐223 expression (B). Data are shown as the mean ± SD.

The expression levels of miR‐223 in the kidneys were determined at various time points (0, 3, 6, 12, and 24 h) post‐RIRI induction. Our data indicated that miR‐223 expression increased shortly after the ischemic event, peaking at 6 h before gradually declining by the 24‐h mark. These findings are shown in Figure 2A. This temporal pattern supports the hypothesis that miR‐223 is involved in the early response to renal I/R injury and may exert anti‐inflammatory effects during this critical period. As miR‐223 levels peaked, NLRP3 levels were significantly reduced, suggesting a regulatory interaction. These results are presented in Figure 2B and support our hypothesis that miR‐223 negatively regulates NLRP3 expression in vivo.

### 3.3 The different levels of miR‐223 in circulation and in kidney tissue in the RIRI model and HK‐2 cells in vitro

**FIGURE 3:**
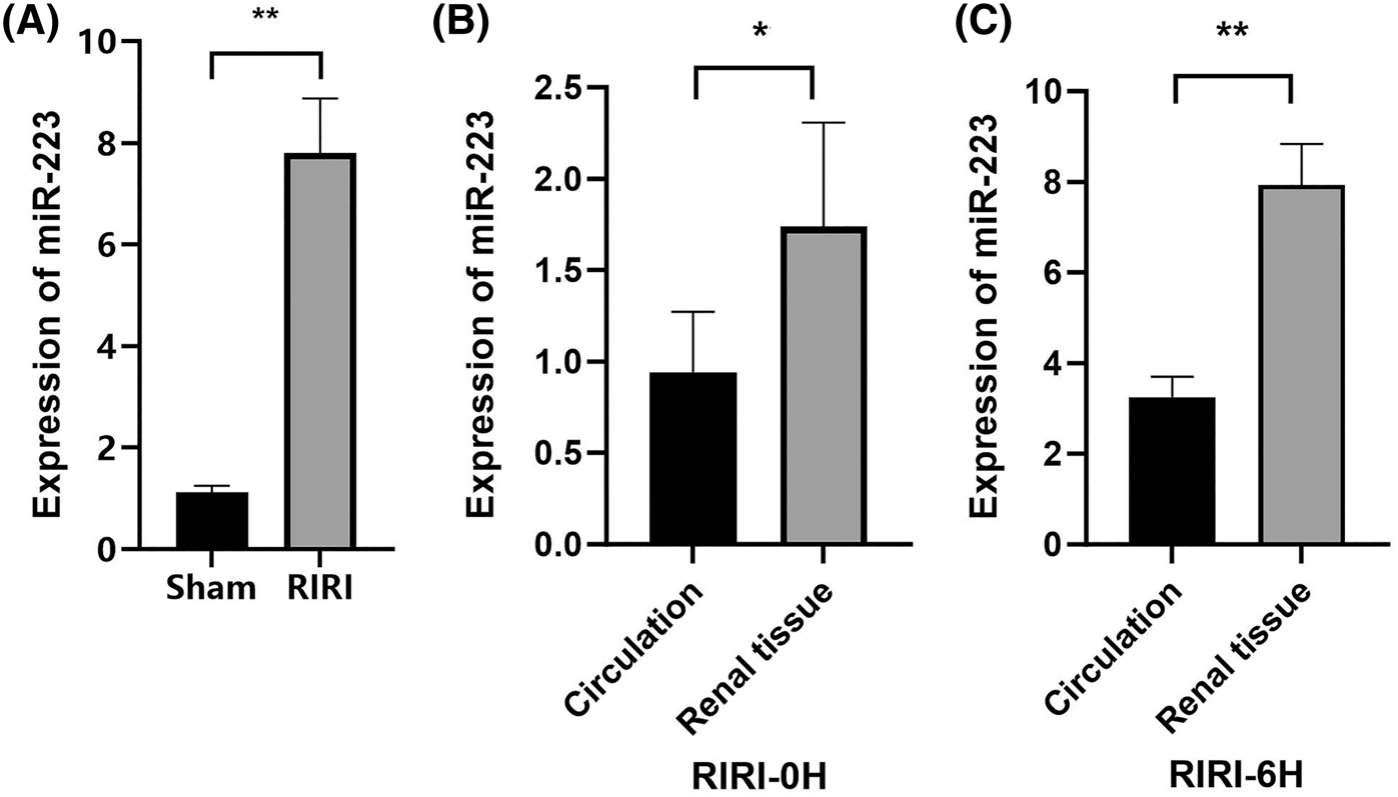
Expression levels of miR‐223 in circulation and in kidney tissue in the RIRI model and HK‐2 cells in vitro. The expression levels of miR‐223 in kidney tissue sections from RIRI mice at 6 h clearly showed that miR‐223 was predominantly localized in the proximal tubules (A). The levels of miR‐223 in circulation (using plasma samples) and kidney tissue (using RT‐qPCR) in RIRI mice at 0 and 6 h, showing that miR‐223 levels are lower in circulation than in kidney tissue (B and C). Data are shown as mean ± SD. *p < 0.05; **p < 0.01.

MiR‐223 was predominantly localized in HK‐2 cells (proximal tubules), thereby validating our choice of HK‐2 cells for the in vitro studies (Figure 3A). Our findings indicated that miR‐223 levels were indeed lower in circulation than in kidney tissue at different times. These data are shown in Figure 3B,C and discussed to clarify the context of miR‐223's systemic versus local expression.

### 3.4 MiR‐223 was decreased and inflammatory cytokines were increased in RIRI mice

**FIGURE 4:**
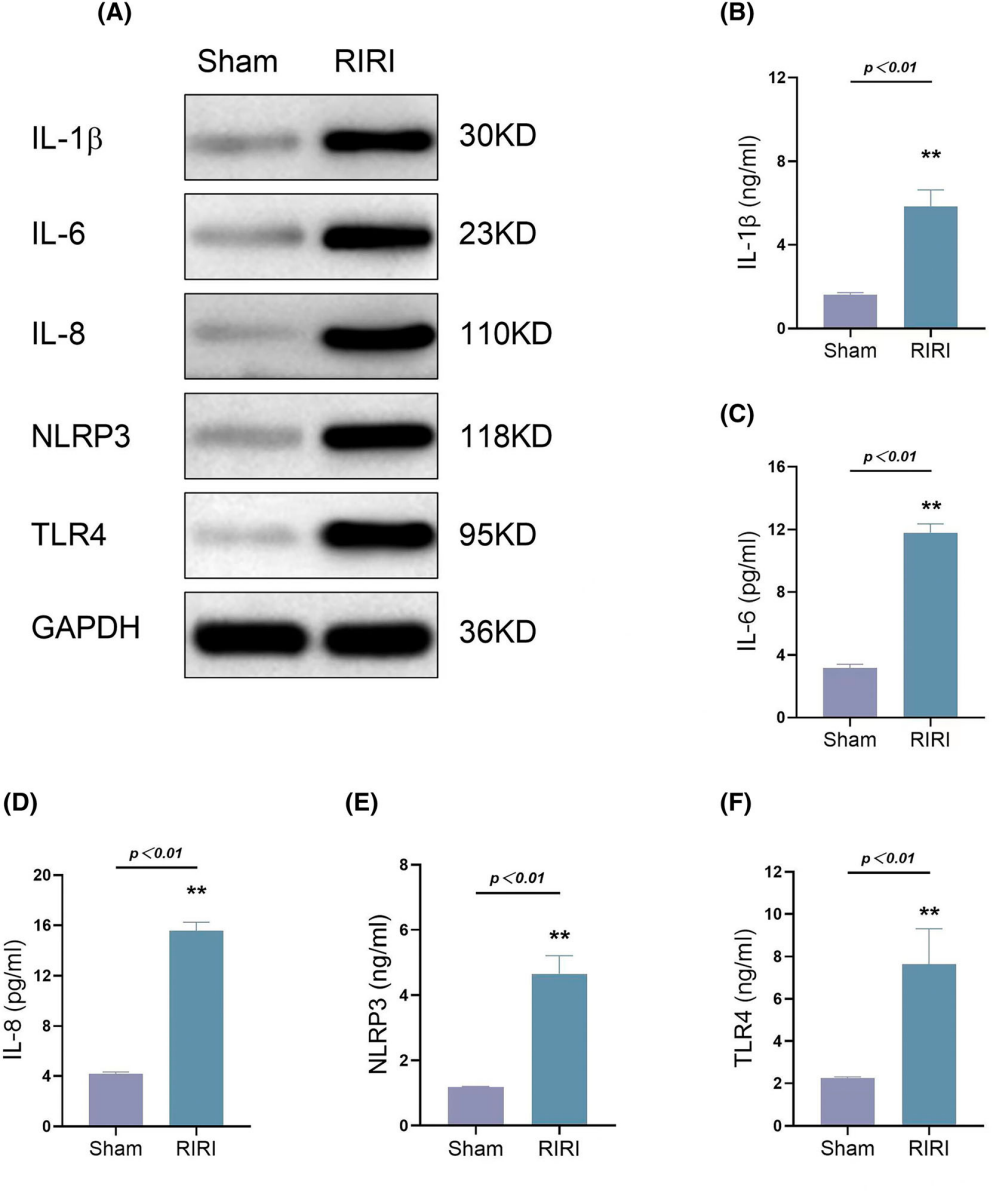
Inflammatory cytokines and NLRP3 and TLR4 expression in the sham‐operation and RIRI group. Western blot assay of lL‐1β, IL‐6, IL‐8, NLRP3, and TLR4 in sham‐operation and RIRI group using kidney tissue (A). Level of lL‐1β, IL‐6, IL‐8, NLRP3, and TLR4 in the sham‐operation and RIRI group via ELISA using plasma (B–F). Data are shown as mean ± SD, *n* = 3; *p < 0.05; **p < 0.01.

The levels of IL‐1β, IL‐6, IL‐8, NLRP3, and TLR4 were more elevated in the RIRI group than in the sham operation group. This trend was confirmed by kidney ELISA and western blotting (Figure 4A–F). Taken together, these results indicate that miR‐223 might be a protective factor in RIRI, as decreasing miR‐223 in RIRI mice resulted in increased activated inflammation, increased expression of inflammatory cytokines, and more damaged kidney tubules.

### 3.5 Relationship between miR‐223 and the TLR4/NF‐κB pathway in RIRI mice

**FIGURE 5:**
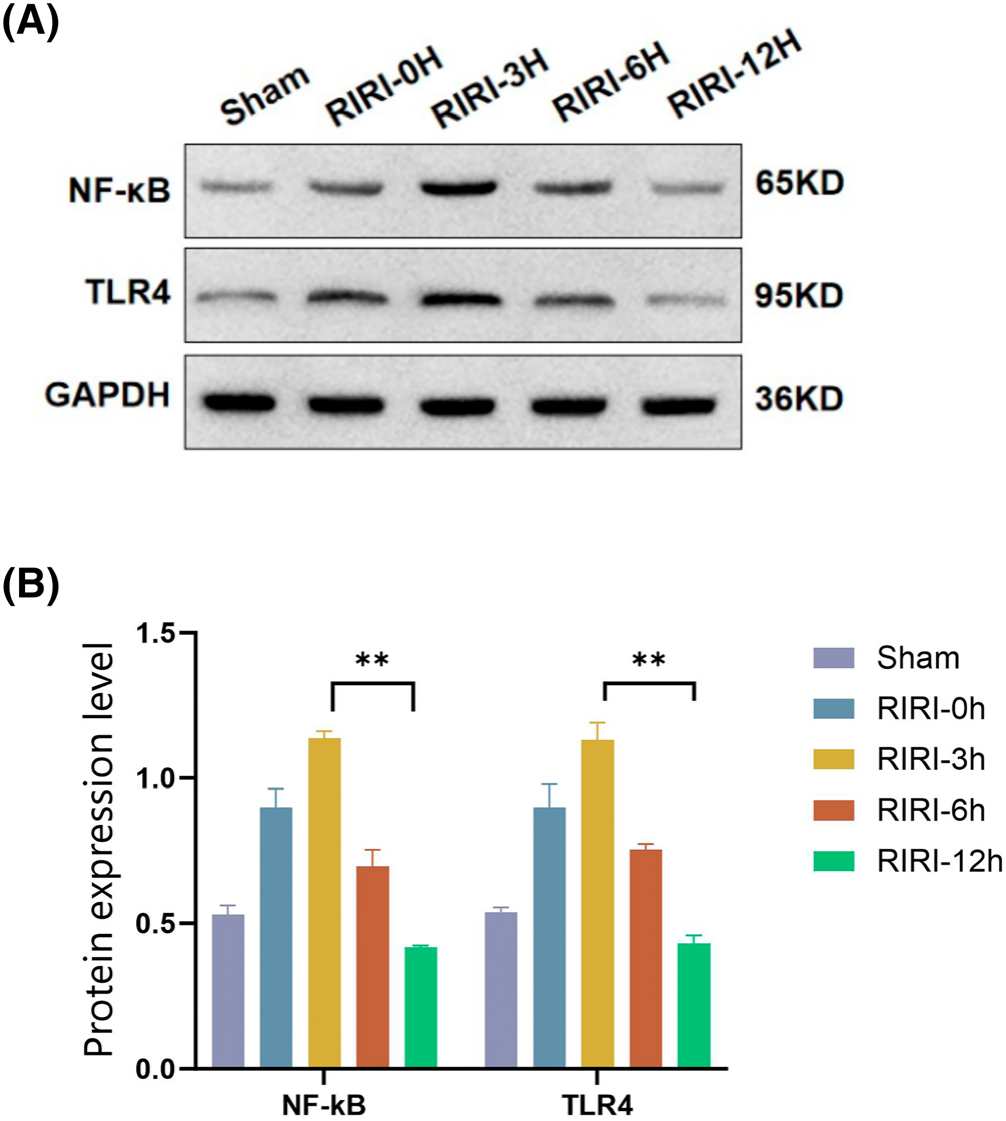
Relationship between miR‐223 and the TLR4/NF‐κB pathway in RIRI mice. Protein levels of TLR4 and NF‐κB in the kidneys of RIRI mice at 0, 3, 6, and 12 h by western blotting (A). Quantitative analysis of NF‐κB and TLR4 protein expression levels in sham and RIRI groups at 0, 3, 6, and 12 h (B). With an increase in miR‐223 expression at 6 h, the protein levels of TLR4 and NF‐κB gradually decreased. Data are shown as the mean ± SD. *p < 0.05; **p < 0.01.

**FIGURE 6:**
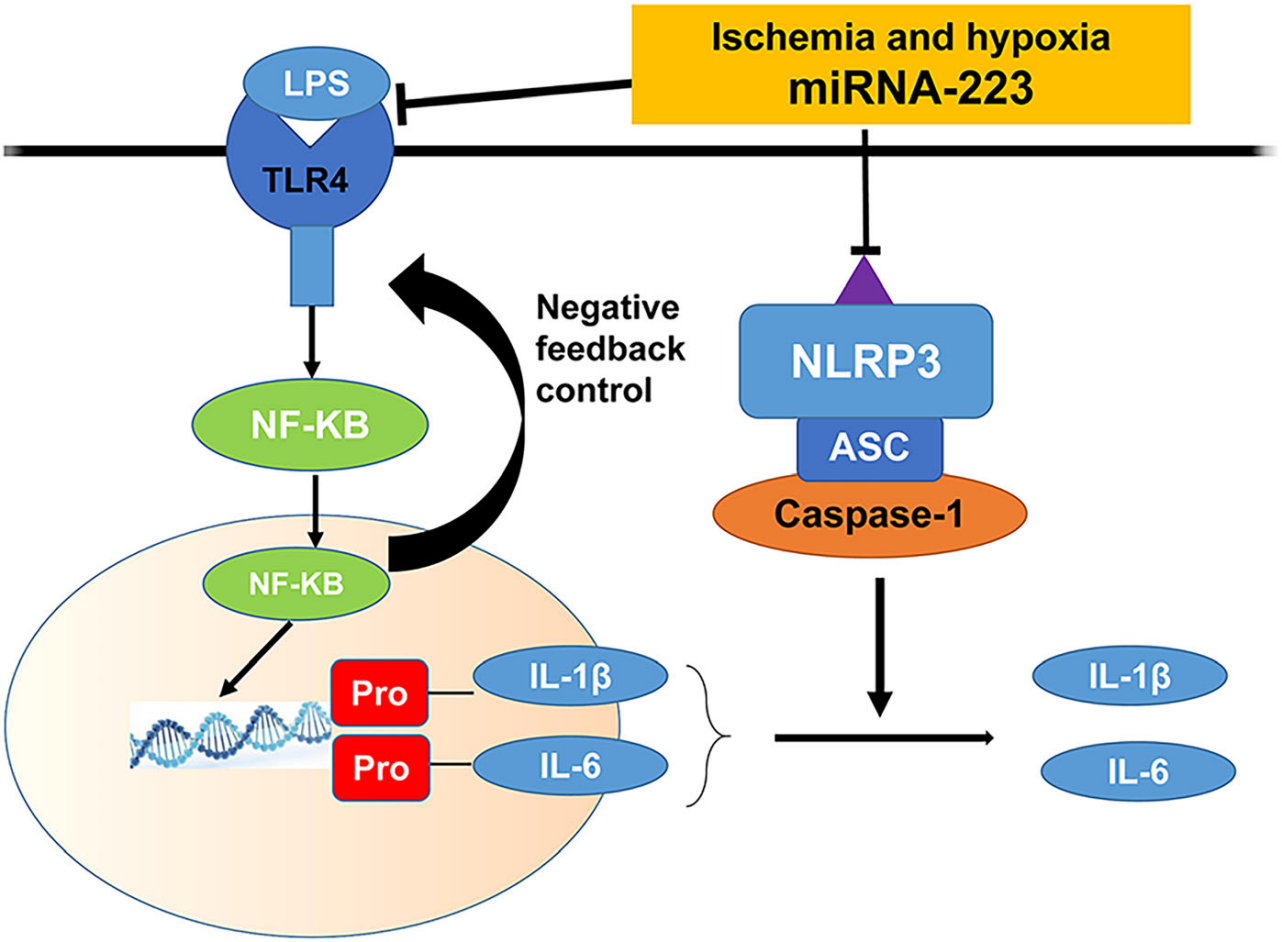
Schematic diagram of miR‐223 regulating the TLR4/NF‐κB pathway.

We measured the expression levels of TLR4 and NF‐κB in the kidneys of RIRI mice at 0, 3, 6, and 12 h, alongside miR‐223. The results showed that miR‐223 overexpression correlated with decreased TLR4 and NF‐κB activity at 6 h, suggesting that miR‐223 may exert anti‐inflammatory effects through this pathway. These findings are shown in Figure 5A,B and a graph of the model pattern is shown in Figure 6.

### 3.6 Extra miR‐223 could alleviate inflammation in RIRI

**FIGURE 7:**
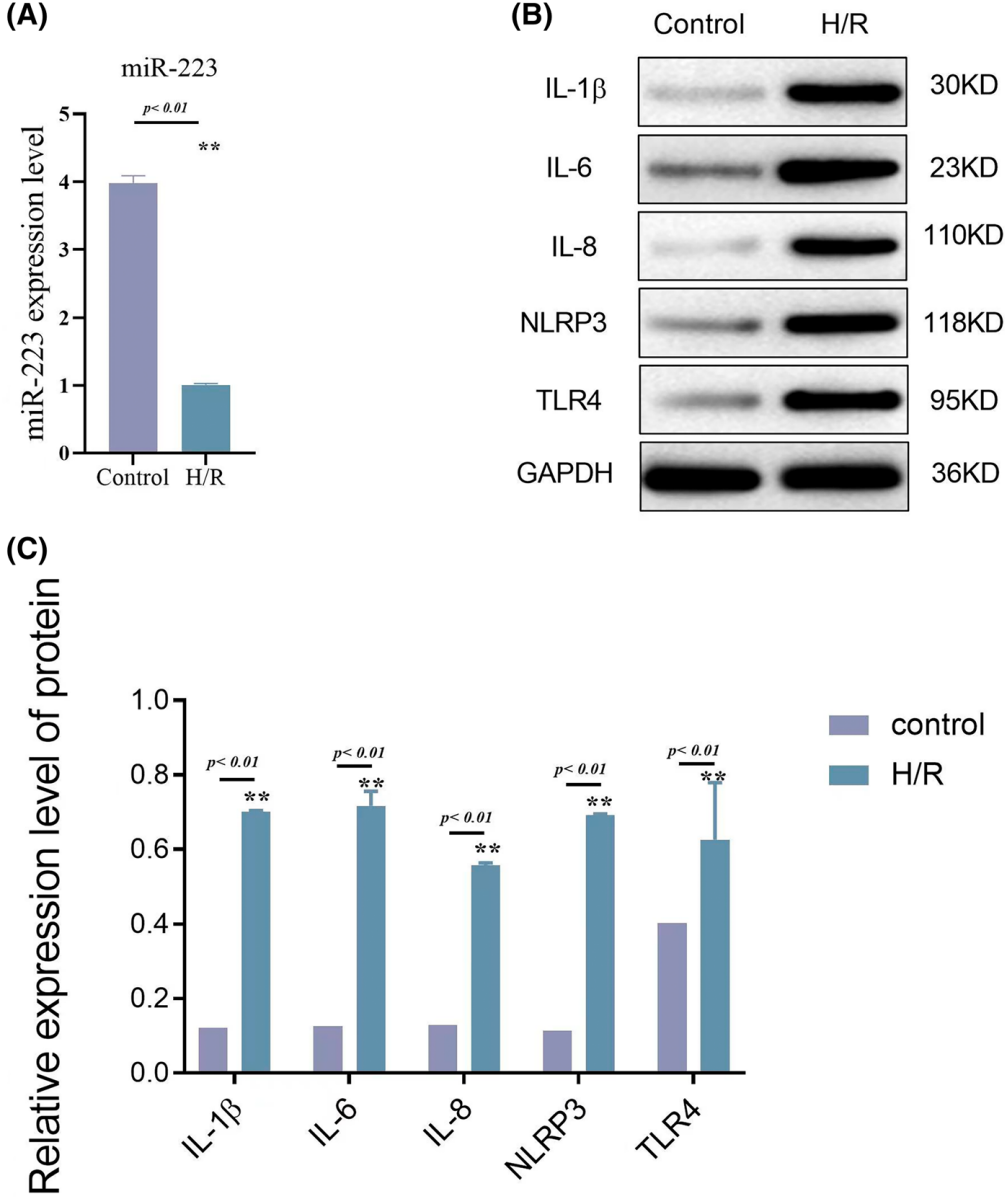
MicroRNA was down‐regulated in H/R compared to control and H/R significantly elevated inflammatory cytokines and NLRP3 and TLR4 in HK‐2 cells at the 0 h mark. RT‐gPCR of miR‐223 in the control and H/R group (A); western blot assay of lL‐1β, IL‐6, IL‐8, NLRP3, and TLR4 expression level in control and H/R group (B), quantitative analysis of lL‐1β, IL‐6, IL‐8, NLRP3, and TLR4 via western blot assay (C). Data are shown as mean ± SD, *n* = 3; *p < 0.05; **p < 0.01.

**FIGURE 8:**
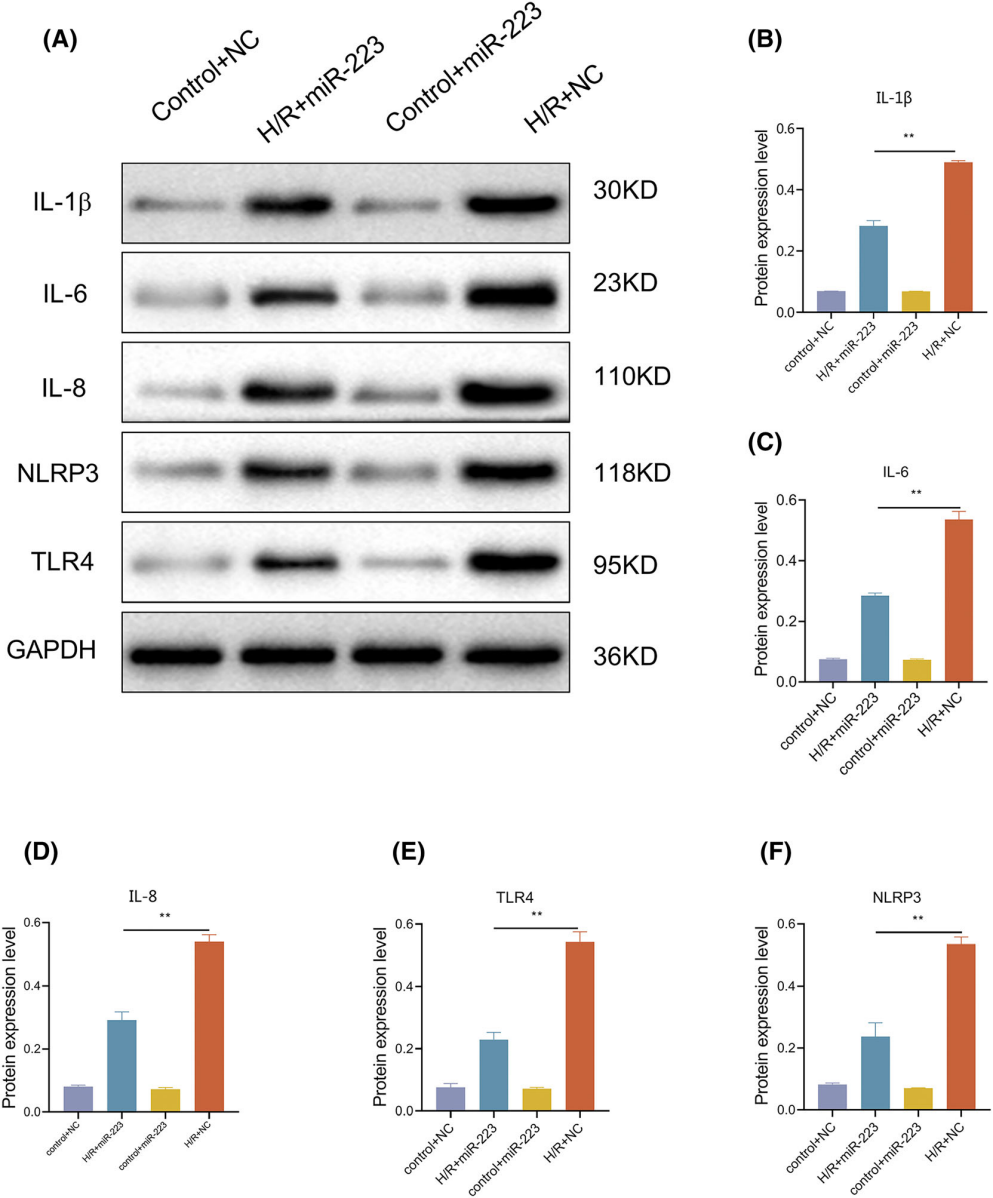
MiR‐223 could alleviate inflammation in H/R. Western blot assay of IL‐1β, IL‐6, IL‐8, NLRP3, and TLR4 in H/R and Control with or without miR‐223 overexpression in HK‐2 cells (A); quantitative analysis of western blot of relative protein (B–F). Data are shown as mean ± SD, *n* = 3; *p < 0.05; **p < 0.01.

**FIGURE 9:**
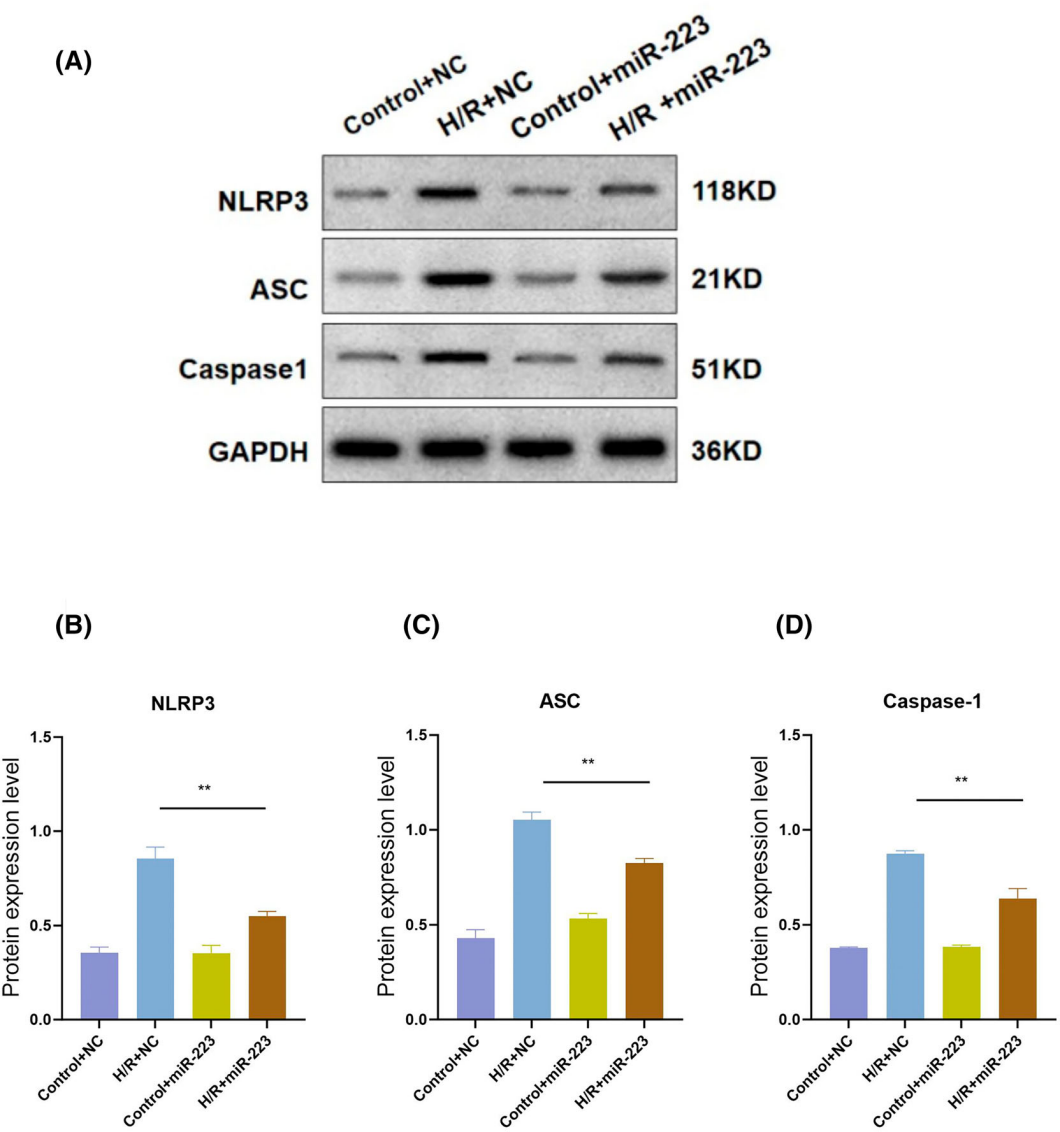
Overexpression of miR‐223 could alleviate inflammation in H/R. Western blot assay of NLRP3, ASC, and Caspase‐1 in H/R and Control with or without miR‐223 overexpression in HK‐2 cells (A); quantitative analysis of western blot of relative protein (B–D). Data are shown as mean ± SD, *p < 0.05; **p < 0.01.

HK‐2 cells were used for the in vitro experiments. After H/R intervention, the levels of miR‐223 and inflammatory cytokines showed trends similar to those in mice (Figure 7A–C). We then upregulated miR‐223 expression in HK‐2 cells. The results showed that extra miR‐223 could significantly alleviate inflammation in H/R cells as levels of IL‐1β, IL‐6, IL‐8, NLRP3, and TLR4 were decreased (Figure 8A–F), indicating the anti‐inflammatory role of miR‐223 in HK‐2 cells. We measured the expression of key inflammasome components, including NLRP3, ASC (apoptosis‐associated speck‐like protein containing a CARD), and caspase‐1, in HK‐2 cells. Our results showed that the overexpression of miR‐223 significantly reduced the activation of the NLRP3 inflammasome, as evidenced by decreased levels of ASC and cleaved caspase‐1. These findings are shown in Figure 9A–D.

### 3.7 MiR‐223 targeted NLRP3 and regulated NLRP3 expression

**FIGURE 10:**
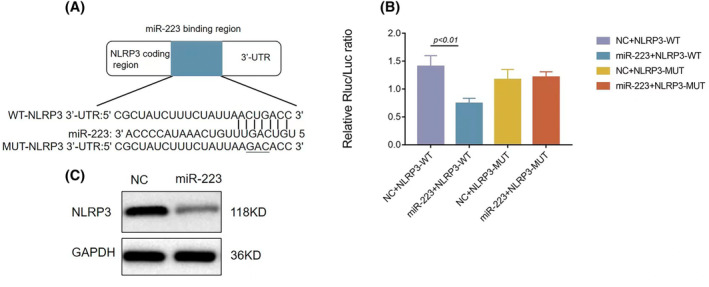
MiR‐223 directly bound to NLRP3 in HK‐2 cells; illustration of plasmids containing wild type (WT) NLRP3 sequence. MiR‐223 binding region with NLRP3 and Mutational (MUT) sequence of NLRP3 (A): Double luciferase reporting results of WT NLRP3 and MUT NLRP3 and miR‐223 (B). Western blot assay of NLRP3 in control and extra miR‐223 cell lines (C). Data are shown as mean ± SD, *n* = 3; *p < 0.05; **p < 0.01.

We searched TargetScan for a possible target gene of miR‐223 and found that NLRP3 was a potential target. We performed a dual‐luciferase reporter assay. The results showed that miR‐223 could bind to the 3′‐UTR of NLRP3 (Figure 10A,B). Western blotting confirmed that in the miR‐223 mimic group, the expression of NLRP3 was down‐regulated compared to the control group (Figure 10C).

### 3.8 MiR‐223 anti‐inflammatory function was dependent on NLRP3

**FIGURE 11:**
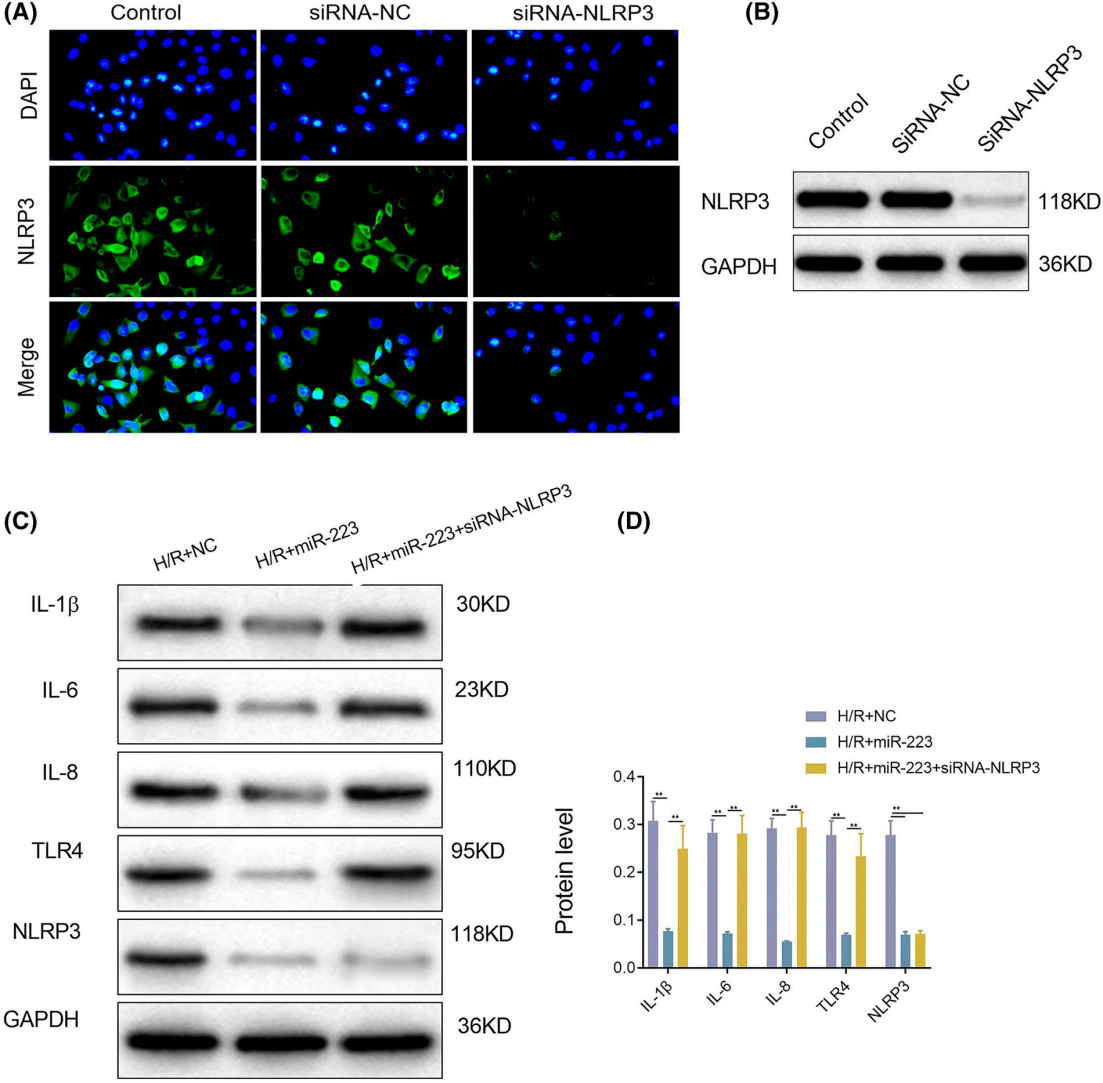
Lock of NLRP3 could abolish the anti‐inflammatory effects of miR‐223. Verification of SiRNA‐NLRP3 in HK‐2 cells, immunofluorescence (A), western blot (B); western blot assay of IL‐1β, IL‐6, IL‐8, NLRP3, and TLR4 in H/R, H/R + miR‐223, and H/R + miR‐223 + SiRNA‐NLRP3 group (C), quantitative analysis of the relative protein in each group (D). Data are shown as mean ± SD.*p < 0.05; **p < 0.01.

We conducted western blotting and immunofluorescence assays to confirm the downregulation of NLRP3 in HK‐2 cells. As shown in Figure 11A,B, the expression level of NLRP3 was significantly lower than that in the control group. As shown in Figure 11C, extra miR‐223 decreased the inflammatory cytokine levels in the H/R group compared to the no extra miR‐223 group. However, the anti‐inflammatory function of miR‐223 in H/R cells was abrogated in the absence of NLRP3 (Figure 11C, the 3rd column). These results suggest that the anti‐inflammatory function of miR‐223 in RIRI is mediated by NLRP3 suppression. The level of NLRP3 was parallel to that of the inflammatory cytokines, and when NLRP3 was downregulated in HK‐2 cells, the protective effects of miR‐223 were suppressed.

## 4 DISCUSSION

In this study, we investigated the role of miR‐223 in RIRI, a major cause of acute renal failure. Our results demonstrated that miR‐223 was significantly downregulated in RIRI and correlated negatively with serum creatinine levels and the severity of tubular injury. We found that miR‐223 exerts anti‐inflammatory effects by targeting the NLRP3 inflammasome, reducing the expression of pro‐inflammatory cytokines such as IL‐1β, IL‐6, and IL‐8. In vitro experiments using HK‐2 cells confirmed that miR‐223 overexpression alleviated inflammation induced by hypoxia/reoxygenation (H/R) treatment, and these effects are dependent on NLRP3. These findings suggest that miR‐223 plays a protective role in RIRI by modulating the inflammatory response via NLRP3 inhibition.

The incidence of RIRI has been increasing recently, accounting for approximately 20% of in‐hospital mortality.^13^ In severe trauma, sepsis, or any other condition that may cause RIRI, patients might experience a rapid urine output decrease and serum creatinine elevation, electrolyte disorders, and even multiple organ dysfunction syndrome (MODS).^14^ Therefore, the early recognition and effective management of IRI are of great importance. In previous studies, miR‐223 was highly involved in organ IRI pathogenesis. Ye et al demonstrated that the miR‐223 level was up‐regulated in a lung IRI model; further experiments found that a high level of miR‐223 could inhibit the expression of hypoxia‐inducible factor‐2α (HIF2α) to repress β‐catenin, which can exacerbate lung IRI. In brain IRI, miR‐223 expression increases in parallel with a decrease in the K^+^‐dependent Na^+^/Ca^2+^ exchanger family (NCKX2). According to a previous study, the miR‐223/NCKX2 axis can worsen neurological function. In the present study, we demonstrated that miR‐223 is an anti‐inflammatory factor in RIRI and that its protective function is dependent on NLRP3.

In RIRI mice, we found that miR‐223 levels in the circulation were relatively low and that the level of miR‐223 was negatively correlated with the level of serum creatinine, suggesting that miR‐223 is involved and might have a protective effect in the pathogenesis of RIRI. We scored the injury to the mouse kidney tubules under a microscope. We found that the severity of the tubules negatively correlated with the level of miR‐223 in the kidney. Moreover, NLRP3 and TLR4 are upregulated under IRI conditions, indicating that NLRP3 and TLR4 interact with miR‐223. NLRP3 is a vital protein for the activation of the NLRP3 inflammasome and triggers a drastic inflammation cascade.^15^ In spine cord injury, miR‐223 binds to NLRP3 and alleviates LPS‐induced inflammation.^16^ TLR4 is a member of the Toll‐like receptor family and is an upstream regulator of NLRP3.^17^, ^18^ In cerebral IRI, TLR4 knockdown ameliorated IRI and suppressed the inflammatory response; notably, this protective function of TLR4 absence was exerted through a decrease in NLRP3.^19^

To further explore the mechanism of action of miR‐223 in RIRI, we conducted in vitro functional experiments using HK‐2 cells. After H/R treatment, miR‐223 levels decreased and pro‐inflammatory cytokine levels increased. Next, we transfected miR‐223 mimics into the cells to determine whether miR‐223 could affect HK‐2 cells pretreated with H/R. As expected, miR‐223 mimic repressed IL‐1β, IL‐6, IL8, NLRP3, and TLR4 in the H/R group, suggesting that miR‐223 might play a protective role in RIRI through repression of inflammation. In previous studies, miR‐223 was shown to be involved in the host immune response and inflammation, including the activation and differentiation of immune cells. NLRP3 is a target of miR‐223 in lung and brain diseases. In LPS‐induced kidney injury, miR‐223 can inhibit the inflammatory response by targeting NLRP3^20^; however, no existing study has demonstrated the function of miR‐223/NLRP3 in RIRI. We used TargetScan and conducted dual luciferase and western blot assays to confirm the binding of miR‐223 to NLRP3 in the experimental cells. Finally, we conducted rescue experiments to determine whether the anti‐inflammatory function of miR‐223 was dependent on NLRP3. As expected, after interfering with NLRP3, the protective function of miR‐223 was abolished, as pro‐inflammatory cytokines were upregulated in the miR‐223 mimic and H/R treatment groups. Indicating that miR‐223 regulates the release of downstream inflammatory factors (IL‐1β, IL‐6, and IL‐8) by binding with NLRP3.

The current study has some limitations. Owing to practical limitations, we did not explore the possible mechanisms of NLRP3 and cell apoptosis or other cellular death programs or signals. In the future, we aim to conduct a series of experiments on this aspect.

MiR‐223 was down‐regulated in RIRI mice and negatively correlated with serum creatinine levels and the severity of tubular injury. MiR‐223 expression was also downregulated in H/R‐pretreated HK‐2 cells. H/R treatment could significantly elevate inflammatory factors IL‐1β, IL‐6, IL‐8, NLRP3, and TLR4 in HK‐2 cells. The extra miR‐223 insert protected HK‐2 cells from H/R injury. NLRP3 was a direct target of miR‐223. Downregulation of NLRP3 abolishes the protective function of miR‐223 under H/R conditions. MiR‐223 and its target NLRP3 have great potential for the treatment of RIRI in the future.

## CONFLICT OF INTEREST STATEMENT

The authors declare no conflicts of interest.
